# An applied study to improve Le-Conte pear productivity and quality using potassium humate with hydrogen peroxide or irradiated licorice extract

**DOI:** 10.1038/s41598-025-16287-9

**Published:** 2025-09-12

**Authors:** Mohamed Farouk Ahmed, Radwan Mohamed Ali, Medhat Ibrahim A. Omar, Noha Eid Eliwa

**Affiliations:** 1 Natural Products Department, National Centre for Radiation Research and Technology, Egyptian Atomic Energy Authority, Cairo, Egypt; 2 Biochemistry Department, Faculty of Agriculture, Al Azhar University, Cairo, Egypt; 3 Agronomy Department (Chemistry Branch), Assiut Faculty of Agriculture, Al Azhar University, Assuit, Egypt

**Keywords:** Le-Conte pear, Potassium humate, Hydrogen peroxide, Irradiated licorice extract, Fire blight, Foliar spray, Biotechnology, Plant sciences

## Abstract

Pears are popular among fruits around the world, but their production faces many challenges, the most important of which is fire blight, which affects productivity and tree quality. Foliar application of organic fertilizers and natural substances could maintain productivity and fruit quality. This study was conducted on fifteen-year-old Le-Conte pear trees to evaluate the feasibility of foliar application of potassium humate, hydrogen peroxide, and gamma-irradiated licorice extract to improve yield, quality, and control fire blight. Data revealed that all individual foliar spray treatments significantly improved the fruit’s physical and chemical characteristics and yield, while the percentage of fire blight infected fruits significantly decreased compared to the control (no spray). Combined sprays improved the above-mentioned parameters more than sole application. Gamma-irradiated licorice gave a more superior effect in most cases, while hydrogen peroxide was more effective in combating the fire blight infection. Combination of high levels of potassium and licorice gave the highest increment in TSS (28.1%), vitamin C (66%), and total sugars (37.5%) with maximum reduction of acidity (64.8%) while high levels of potassium and hydrogen peroxide gave the highest yield (85.3%) and maximum reduction of fire-blighted fruits (83.7%). In conclusion, Le-Conte pear trees productivity and quality can be increased, while fire blight affected fruits can be decreased, by using a foliar spray of potassium humate with hydrogen peroxide or irradiated licorice extract.

## Introduction

Pears (*Pyrus communis L.*), which belongs to the Rosaceae family, are among the most significant, well-known, and lucrative fruits in the world. In terms of production, it is the fifth-ranked deciduous fruit crop worldwide, with a total production of 23.73 million tons from 1.4 million hectares in 2019^1^. According to the most recent market data, Egypt has consistently produced an annual pear production of approximately 75,000 metric tons with a harvested area of about 5600 hectares and an average yield of 13 tons per hectare. This reflects a minor drop of 1.8% from the prior year^2^. This kind of fruit is cultivated mainly for its high usability, it is initially enjoyed as a popular fresh fruit and, because of its high content of "primary and secondary metabolites," which are crucial for human nutrition and health, can also be processed into a wide range of products^3^. The primary pear cultivar grown in Egypt is the Le-Conte pear which is a hybrid of *Pyrus communis* and *Pyrus serotina*. However, over the previous few decades, the cultivated area of pears varied significantly due to the fire blight outbreak^4^. Fire blight, caused by the pathogenic bacteria *Erwinia amylovora*, is the most destructive factor that greatly affects the growth, productivity, and quality of pear trees. These bacteria infect pears as well as most other members of the Rosacea family and are currently found in more than 50 countries, including Egypt^5^. Recently, environmental challenges have attracted attention because they have a major impact on fire blight infection and fruit quality. Natural products are viewed as an alternative to many synthetic materials used as plant nutrition because of their accessibility, affordability, and minimal potential for causing pollution or degrading soil and land^6^.

It has been established that humic acid (HA), an organically charged biostimulant, has a positive impact on every aspect of agriculture. Beginning with the soil, humic acid addition enhances the soil’s chemical, biological, and physical characteristics^7^. Additionally, Canellas and Olivares^8^ reported that HA improved a number of physiological processes in plants, such as enhancing permeability of cell membranes, nutrient absorption, triggering the synthesis of chlorophyll pigment, and facilitating the assembly of sugars, amino acids, and enzymes. One of HA’s unique qualities is that it makes plants more disease-resistant, which improves plant health. Additionally, it greatly reduces the stresses of high salinity and temperature that can lead to toxicity, which gives the plant a degree of resistance and benefits the continuation of essential processes^9,10^. Apart from these benefits, some research has demonstrated that HA spraying improved fruit size, weight, total soluble solids, and vitamin C contents, as demonstrated in guava^11^ and apricots^12^.

In recent decades, interest in hydrogen peroxide (H_2_O_2_), the most stable reactive oxygen species with a relatively long half-life, has grown significantly as a critical regulator of numerous physiological and biochemical processes in plants, both under stress and under normal circumstances^13^. The plant is sensitive to H_2_O_2_, and while a high concentration can stress the plant to the point of death, low and normal concentrations aid in the plant’s ability to withstand a variety of stresses^14^. Low-concentration foliar application of H_2_O_2_ not only promotes plant growth, enzymatic antioxidant activities, fruit yield, and quality^15^, but it also plays a vital role in combating plant pathogens like wheat leaf rust^16^ and barley net blotch disease^17^. Furthermore, non-host resistance mechanisms in cereal and legume plants against incompatible pathogens greatly depend on an early increase in H_2_O_2_^18^.

Plant extracts are one kind of biostimulant that agriculture has recently adopted on a large scale. Licorice root (*glycyrrhiza glabra)*, a member of the Leguminoseae family of plants that grows widely throughout the world, including Egypt, could be used as a safe substitute for chemical growth regulators^19^. More than 100 necessary substances, such as flavonoids, phenolic compounds, minerals, proteins, amino acids, monosaccharaides, several vitamins, lignin, tannins, and mevalonic acid that is involved in the synthesis of gibberellins, are found in licorice root extract^20^. Because of all these components, licorice helps with most of the essential functions of the plant, including promoting cell division and cellulose enzyme activity, which in turn promotes plant growth. Additionally, it improves the plant’s capacity to reduce rates of transpiration and water loss, preserving cell tension^21^. Spraying potato plants^22^ and pear trees^4^ with foliar licorice root extract enhanced the plants’ growth and leaf mineral contents, ultimately increasing their marketable yield.

Ionizing radiation exposure, including electron beam, X-rays, and gamma rays, is one feasible method of reducing the microbiological load of medicinal herbs^23^. However, depending on the radiation dose and herb type, the effect of radiation varies from negligible to minimal on the volatile oil content and overall yield of herbal extracts^24,25^ and may result in a significant increase in some bioactive ingredients^26^. Licorice exposed to gamma radiation showed a significant increase in the total yield of volatile compounds^27^, as well as in the concentration of maltose and glycyrrhezinic acid in the resultant solutions^28^.

Therefore, the purpose of this study was to evaluate the feasibility of foliar application of potassium humate, hydrogen peroxide, and gamma-irradiated licorice extract to improve production, quality, and fire blight infection control of Le-Conte pear. We hypothesize that, foliar spraying Le-Conte pear trees with potassium humate, hydrogen peroxide, and gamma-irradiated licorice extract will boost fruit quality and yield while reducing fire blight. The novel aspect of this study is the application of hydrogen peroxide, potassium humate, and gamma-irradiated licorice extract as eco-friendly foliar treatments. This report is the first to look at the use of environmentally friendly compounds to improve the fruit quality and productivity of pear trees in Egypt and prevent fire blight.

## Results

### Fruit physical characteristics

All treatments enhanced the Le-Conte pear fruits’ diameter, length, firmness, and size as compared to the control (Table 1). The foliar application of potassium humate, hydrogen peroxide, or irradiated licorice extract alone or in combination greatly raised the previously stated parameters. When those treatments were taken at the higher level, their effects became noticeably stronger. In comparison to hydrogen peroxide, the increment effect of irradiated licorice extract was greater. The highest values of fruit size, length, diameter, and firmness were obtained when high level of potassium humate combined with a high level of irradiated licorice extract or hydrogen peroxide. Sole foliar application of the high level of potassium humate and hydrogen peroxide as well as irradiated licorice extract greatly improved the fruit shape index. While all combined treatments resulted in a notable increase in the fruit shape index, high levels of irradiated licorice extract and hydrogen peroxide had the greatest increase. The greatest improvement was found when combining potassium humate with either hydrogen peroxide or irradiated licorice extract at their high levels.

**Table 1:** The effect of potassium humate, hydrogen peroxide, and irradiated licorice extract on some physical characteristics of Le-Conte pear fruits.

	Fruit size (cm^3^)		Fruit length (cm)		Fruit diameter (cm)		Fruit shape index (%)		Fruit firmness (N)	
	1st	2nd	1st	2nd	1st	2nd	1st	2nd	1st	2nd
Control	156 h	162 j	7.33 i	7.53 h	6.00 f	6.10 h	1.222 h	1.234 i	13.4 h	13.5 h
K1	168 g	173 i	8.07 h	8.13 g	6.50 de	6.52 fg	1.242 gh	1.247 hi	14.4 g	14.5 g
K2	178 f	181 g	8.50 fg	8.47 g	6.83 c	6.77 ef	1.245 g	1.251 h	14.7 f	15.2 e
H1	171 g	178 h	8.17 h	8.20 g	6.60 cde	6.57 efg	1.238 gh	1.248 hi	14.8 f	14.8 f
H2	185 e	188 f	8.73 ef	8.90 f	6.50 de	6.60 efg	1.343 d	1.348 ef	15.2 e	15.5 d
L1	178 f	183 g	8.30 gh	8.23 g	6.43 e	6.33 gh	1.291 f	1.300 g	15.3 e	15.7 d
L2	189 d	196 de	8.73 ef	9.53 e	6.63 cde	7.13 bc	1.317 e	1.337 f	16.3 c	16.7 c
K1H1	191 d	194 e	8.93 de	9.20 f	6.67 cde	6.83 de	1.339 d	1.347 ef	15.3 e	15.6 d
K1H2	196 c	198 cd	9.17 d	9.67 de	6.73 cd	7.07 cd	1.363 c	1.368 cd	16.9 b	17.2 b
K1L1	189 d	193 e	8.93 de	9.13 f	6.67 cde	6.77 ef	1.339 d	1.349 def	16.3 cd	16.7 c
K1L2	201 ab	206 b	10.07 b	10.47 b	7.23 ab	7.40 b	1.393 a	1.415 a	17.5 a	17.7 a
K2H1	196 c	199 c	9.00 d	9.97 cd	6.73 cd	7.37 b	1.337 d	1.353 de	16.1 d	16.6 c
K2H2	202 ab	208 ab	9.80 c	10.63 ab	7.17 ab	7.70 a	1.367 bc	1.381 bc	17.5 a	17.8 a
K2L1	199 bc	201 c	9.83 c	10.10 c	7.10 b	7.27 bc	1.385 ab	1.389 b	16.9 b	17.3 b
K2L2	204 a	209 a	10.30 a	10.87 a	7.37 a	7.70 a	1.398 a	1.412 a	17.5 a	18.0 a

Means with different letters are significantly different at p < 0.05

*K1* potassium humate at 1%, *K2* potassium humate at 2%, *H1* hydrogen peroxide at 1 ml/L, *H2* hydrogen peroxide at 2 ml/L, *L1* gamma-irradiated licorice at 4 g/L, and *L2* gamma-irradiated licorice at 8 g/L

### Yield characteristics

The findings presented in Table 2 demonstrated a significant increase in Le-Conte pear fruit number/tree, fruit weight, and yield/tree upon foliar application of potassium humate, hydrogen peroxide, or irradiated licorice extract in comparison to the control. The enhancement effect became stronger when the level of these treatments increased. Either hydrogen peroxide or irradiated licorice extract produced a more favorable impact than potassium humate, with irradiated licorice extract being chosen in most cases. Also, the aforementioned characteristics were greatly boosted by all combined foliar sprays. The combinations of hydrogen peroxide or irradiated licorice extract with potassium humate at their high levels were better than their low levels. The highest rate of increment in fruit number and yield were obtained by combined high levels of potassium humate and hydrogen peroxide treatment (43.9% and 85.3%, respectively) while the highest rate of increment in fruit weight was obtained by combined high level of potassium humate and irradiated licorice treatment (30.1%). The number of fire blighted fruits and, hence, the percentage of fire blighted fruits were dramatically decreased when potassium humate, hydrogen peroxide, or irradiated licorice extract were sprayed individually compared to the control. Hydrogen peroxide, in particularly at the high level where the proportion of fire-blighted fruits was 1.30% and 1.28% in both seasons, respectively, proved to be the most successful solitary application. All combined sprays resulted in a significant decrease in the number of fire-blighted fruits, particularly for sprays with a high concentration of hydrogen peroxide. When potassium humate at the high level was combined with hydrogen peroxide at the high level, the lowest percentage of fire-blighted fruits was obtained (1.02% and 0.94% for both seasons, respectively). This was followed by potassium humate at the low level combined with hydrogen peroxide at the high level (1.14% and 1.05% for both seasons, respectively).

**Table 2:** The effect of potassium humate, hydrogen peroxide, and irradiated licorice extract on some yield characteristics and fire blight of Le-Conte pear fruits.

	Fruit number/tree		Fruit weight (g)		Yield/ tree (Kg)		Number of fire blight infected fruits/ tree		Percentage of fire blight infected fruits (%)	
	1st	2nd	1st	2nd	1st	2nd	1st	2nd	1st	2nd
Control	157 k	162 l	155 h	161 j	24.4 j	26.0 j	14.00 a	13.60 a	8.903 a	8.412 a
K1	167 j	170 k	167 g	172 i	27.9 i	29.2 i	13.63 ab	12.97 b	8.163 b	7.627 b
K2	177 i	181 j	177 f	180 g	31.3 h	32.6 h	13.00 b	12.77 b	7.360 c	7.054 c
H1	182 h	190 i	170 g	177 h	30.9 h	33.6 g	6.00 ef	5.20 e	3.296 ef	2.737 e
H2	197 ef	203 g	184 ab	187 f	36.3 f	38.0 e	2.57 i	2.60 g	1.302 j	1.280 h
L1	192 g	202 g	176 f	182 g	33.8 g	36.8 f	7.50 c	7.00 c	3.914 d	3.459 d
L2	201 de	209 f	187 d	194 de	37.7 de	40.6 d	6.10 ef	5.73 d	3.029 fg	2.748 e
K1H1	194 fg	198 h	190 d	193 e	36.8 ef	38.3 e	5.50 fgh	4.87 ef	2.838 gh	2.456 f
K1H2	215 bc	220 d	195 c	197 cd	41.9 b	43.3 c	2.43 i	2.30 g	1.135 j	1.046 hi
K1L1	204 d	208 f	188 d	192 e	38.4 cd	39.9 d	7.10 cd	6.90 c	3.482 e	3.317 d
K1L2	213 c	226 c	200 ab	204 b	42.5 b	46.2 b	5.73 fg	4.97 e	2.695 ghi	2.198 g
K2H1	202 de	205 g	194 c	198 c	39.3 c	40.5 d	4.97 h	4.37 f	2.461 hi	2.130 g
K2H2	225 a	234 a	200 ab	206 ab	45.1 a	48.3 a	2.30 i	2.20 g	1.022 j	0.940 i
K2L1	212 c	216 e	197 bc	200 c	41.9 b	43.2 c	6.53 de	5.90 d	3.079 fg	2.727 e
K2L2	218 b	231 b	203 a	208 a	44.3 a	47.9 a	5.20 gh	4.73 ef	2.390 i	2.051 g

Means with different letters are significantly different at p < 0.05.

*K1* potassium humate at 1%, *K2* potassium humate at 2%, *H1* hydrogen peroxide at 1 ml/L, *H2* hydrogen peroxide at 2 ml/L, *L1* gamma-irradiated licorice at 4 g/L, and *L2* gamma-irradiated licorice at 8 g/L.

### Fruit chemical characteristics

Table 3 showed how the TSS, acidity, TSS/acid ratio, and vitamin C content of Le-Conte pear fruit were affected by foliar application of potassium humate, hydrogen peroxide, or irradiated licorice extract. The foliar application of potassium humate, hydrogen peroxide, or irradiated licorice extract individually resulted in a considerable rise in fruit TSS, TSS/acid ratio, and vitamin C content compared to the control except potassium humate’s low level, which had no apparent effect on the TSS/acid ratio. As the level of the foliar-sprayed treatments— potassium humate, hydrogen peroxide, or irradiated licorice extract— increased, the rate of increment in the previously mentioned characteristics increased. In addition, it was discovered that foliar spraying of irradiated licorice extract had the greatest rate of improvement (13.77, 13.40; 35.94, 36.92; 3.22, 3.24 for both seasons for TSS, TSS/acid ratio, and vitamin C content, respectively), followed by hydrogen peroxide and potassium humate. The TSS, TSS/acid ratio, and vitamin C content all significantly increased as a result of the combination sprays; sprays with high treatment levels were more effective over those with lower levels, and irradiated licorice extract was preferred over hydrogen peroxide and potassium humate. Foliar spraying of different treatments and levels, either individually or in combination, led to a significant reduction in the acidity when compared to the control. Once more, when the level of foliar spray treatments increases, so does the rate of decrease in acidity. Additionally, the highest reduction in acidity was observed with irradiated licorice extract-containing sprays, which was followed by hydrogen peroxide and potassium humate.

**Table 3:** The effect of potassium humate, hydrogen peroxide, and irradiated licorice extract on some chemical characteristics of Le-Conte pear fruits.

	TSS (%)		Acidity (%)		TSS/acid ratio		Vitamin C (mg/100 g)	
	1st	2nd	1st	2nd	1st	2nd	1st	2nd
Control	11.13 i	11.37 i	0.64 a	0.58 a	17.34 i	19.6 i	2.14 k	2.21 h
K1	11.63 h	11.77 h	0.59 b	0.54 b	19.72 hi	21.8 hi	2.22 j	2.25 h
K2	12.00 g	12.03 g	0.53 d	0.51 c	22.51 h	23.6 h	2.31 i	2.42 g
H1	12.20 g	12.10 g	0.56 c	0.54 b	21.81 h	22.41 hi	2.34 i	2.45 g
H2	12.83 f	13.10 f	0.39 g	0.40 e	32.93 ef	32.76 fg	2.57 g	2.71 e
L1	12.73 f	13.07 f	0.41 f	0.39 e	31.06 fg	33.52 ef	2.81 f	3.00 d
L2	13.77 cd	13.40 e	0.38 g	0.36 f	35.94 e	36.92 de	3.22 d	3.24 c
K1H1	12.80 f	13.00 f	0.45 e	0.44 d	28.34 g	29.37 g	2.42 h	2.48 g
K1H2	13.23 e	13.60 d	0.30 h	0.29 g	44.13 d	47.55 c	3.07 e	3.18 c
K1L1	13.60 d	13.37 e	0.38 g	0.35 f	35.82 e	38.21 d	3.12 e	3.22 c
K1L2	14.03 b	14.27 b	0.28 i	0.22 h	49.64 c	64.08 b	3.33 c	3.43 b
K2H1	12.90 f	13.10 f	0.38 g	0.36 f	34.06 ef	36.41 de	2.53 g	2.61 f
K2H2	13.90 bc	14.10 c	0.25 j	0.23 h	56.49 b	62.57 b	3.43 b	3.46 b
K2L1	13.90 bc	14.03 c	0.31 h	0.28 g	44.42 d	49.63 c	3.35 c	3.51 b
K2L2	14.33 a	14.50 a	0.23 k	0.20 i	63.62 a	71.36 a	3.54 a	3.68 a

Means with different letters are significantly different at p < 0.05.

*K1* potassium humate at 1%, *K2* potassium humate at 2%, *H1* hydrogen peroxide at 1 ml/L, *H2* hydrogen peroxide at 2 ml/L, *L1* gamma-irradiated licorice at 4 g/L, and *L2* gamma-irradiated licorice at 8 g/L.

The impact of foliar application of potassium humate, hydrogen peroxide, and irradiated licorice extract on total, reduced, and non-reduced sugar content of Le-Conte pear fruits was illustrated in Table 4. Foliar application of potassium humate, hydrogen peroxide, and irradiated licorice extract, either separately or in combination, had a favorable effect on the total and reduced sugar content compared to the control. Irradiated licorice extract was associated with higher increases in total and reduced sugar content among sole applications. Foliar spraying of potassium humate and irradiated licorice extract had a higher positive impact on total and reduced sugar content compared to potassium humate and hydrogen peroxide. For non-reduced sugars, it showed a significant reduction when potassium humate, hydrogen peroxide, or irradiated licorice extract was foliar applied individually or in combination as compared to the control.

**Table 4:** The effect of potassium hamate, hydrogen peroxide, and irradiated licorice extract on total, reduced and non-reduced sugars content of Le-Conte pear fruits.

	Total sugars (g/100 g FW)		Reduced sugars (g/100 g FW)		Non-reduced sugars (g/100 g FW)	
	1st	2nd	1st	2nd	1st	2nd
Control	8.183 l	8.500 k	5.100 l	5.327 j	3.083 ab	3.173 a
K1	8.440 k	8.917 j	5.373 k	5.900 i	3.067 ab	3.017 c
K2	9.277 j	9.440 i	6.273 j	6.427 h	3.003 c	3.013 c
H1	9.437 i	9.350 i	6.473 i	6.427 h	2.963 de	2.923 de
H2	10.190 g	10.300 g	7.100 g	7.400 f	3.090 a	2.900 e
L1	10.870 e	10.800 d	7.973 e	8.000 d	2.897 f	2.800 f
L2	10.967 de	10.600 e	8.000 de	8.003 d	2.967 de	2.597 h
K1H1	9.790 h	10.020 h	6.927 h	7.027 g	2.863 fg	2.993 c
K1H2	10.423 f	10.937 c	7.373 f	8.027 d	3.050 b	2.910 e
K1L1	11.010 cd	10.800 d	8.073 cd	8.100 c	2.937 e	2.700 g
K1L2	11.110 bc	11.307 b	8.217 b	8.250 b	2.893 f	3.057 b
K2H1	10.123 g	10.427 f	7.133 g	7.500 e	2.990 cd	2.927 de
K2H2	10.933 de	11.193 b	8.100 c	8.300 b	2.833 g	2.893 e
K2L1	11.150 b	11.200 b	8.200 b	8.250 b	2.950 e	2.950 d
K2L2	11.400 a	11.540 a	8.527 a	8.627 a	2.873 f	2.913 e

Means with different letters are significantly different at p < 0.05

*K1* potassium humate at 1%, *K2* potassium humate at 2%, *H1* hydrogen peroxide at 1 ml/L, *H2* hydrogen peroxide at 2 ml/L, *L1* gamma-irradiated licorice at 4 g/L, and *L2* gamma-irradiated licorice at 8 g/L.

The results in Table 5 demonstrate the effects of spraying potassium humate, hydrogen peroxide, and irradiated licorice extract on the potassium, zinc, and iron levels of Le-Conte pears. The K% in Le-Conte pear fruits was dramatically increased by each individual foliar spraying with potassium humate, hydrogen peroxide, and irradiated licorice extract. The irradiated licorice extract or the high hydrogen peroxide level showed a significant, superior effect over the others. For combination sprays, potassium humate combined with hydrogen peroxide or irradiated licorice extract significantly raised the fruit K%. Spraying potassium humate at the high level with either of the irradiated licorice extract levels, the high hydrogen peroxide level, or potassium humate at the low level with the high irradiated licorice extract level produced the greatest K% values. All foliar treatments, whether sprayed singly or in combination, significantly increased the Zn and Fe content compared to the control. Sole foliar spraying of irradiated licorice extract led to a more notable increase in Zn and Fe content, followed by hydrogen peroxide, then potassium humate, with a preference for the high level of each substance over the low one. The fruit content of Zn and Fe increased significantly as a result of all combined sprays, while the ones containing potassium humate and irradiated licorice extract were preferred over the others. When high levels of irradiated licorice extract and potassium humate were sprayed, the greatest values for Zn and Fe content were achieved (41.0, 42.0, 141.0, and 141.3 for Zn and Fe in both seasons, respectively).

**Table 5:** The effect of potassium humate, hydrogen peroxide, and irradiated licorice extract on K (%), Zn (ppm), and Fe (ppm) content of Le-Conte pear fruits.

	Fruit K (%)		Fruit Zn (ppm)		Fruit Fe (ppm)	
	1st	2nd	1st	2nd	1st	2nd
Control	0.90 g	1.00 g	19.00 l	20.00 k	108.0 j	110.0 j
K1	1.08 f	1.12 f	22.00 k	21.00 j	110.3 i	111.0 j
K2	1.15 de	1.17 def	26.00 j	26.33 i	123.0 h	121.7 i
H1	1.12 ef	1.14 ef	28,00 i	28.67 h	129.0 g	128.0 h
H2	1.19 bcd	1.21 bcd	35.00 f	35.33 e	133.0 e	133.7 f
L1	1.22 bc	1.25 b	37.00 d	38.00 c	136.7 cd	137.7 de
L2	1.23 b	1.26 b	38.00 c	38.67 c	138.0 bc	139.0 bc
K1H1	1.17 cde	1.19 cde	31.33 h	32.33 g	131.0 f	130.7 g
K1H2	1.23 b	1.25 b	36.00 e	36.67 d	136.0 d	136.7 e
K1L1	1.25 b	1.27 b	38.00 c	38.67 c	138.0 bc	138.7 cd
K1L2	1.37 a	1.39 a	39.00 b	40.00 b	139.0 b	140.0 b
K2H1	1.21 bc	1.23 bc	33.00 g	33.67 f	134.0 e	134.3 f
K2H2	1.36 a	1.38 a	37.00 d	37.67 c	137.0 cd	137.7 de
K2L1	1.34 a	1.38 a	39.00 b	39.67 b	139.0 b	139.7 bc
K2L2	1.39 a	1.41 a	41.00 a	42.00 a	141.0 a	141.3 a

Means with different letters are significantly different at p < 0.05.

*K1* potassium humate at 1%, *K2* potassium humate at 2%, *H1* hydrogen peroxide at 1 ml/L, *H2* hydrogen peroxide at 2 ml/L, *L1* gamma-irradiated licorice at 4 g/L, and *L2* gamma-irradiated licorice at 8 g/L.

## Discussion

Research on Le-Conte pear production and fire blight treatments is essential given the increasing demand for new standards for environmentally friendly farming techniques globally due to climate change and future plans for sustainable fruit farming to improve fruit quality and productivity. Two of the risk factors for Erwinia amylovora infection are climatic: rainfall during warm periods and temperatures above 15°C during flowering, which enhance the pathogen’s capacity to multiply to infection-causing levels^29^. In order to improve absorption of the sprayed treatments, foliar spraying of hydrogen peroxide and irradiated licorice extract was selected for February and March, when buds start to appear. Additionally, April and May were selected because rising temperatures encourage the spread of fire blight.

Our study discovered that foliar spraying Le-Conte pear trees with potassium humate improved the physical and chemical properties of the fruits as well as their yield qualities, while also dramatically reducing the proportion of fire-blighted pears. Accordingly, Mosa et al.^30^ discovered that applying 5% or 4% HA to Le-Conte pear trees significantly increased the fruits’ size, length, diameter, firmness, and weight, but had little effect on the fruit shape index. There was a noticeable rise in the ratio of TSS to acidity, total sugars, and soluble solids. Furthermore, 4% HA produced the greatest reduction in fruit acidity, whereas 5% HA significantly raised the quantity of reducing and non-reducing sugars in pear fruits. Likewise, the physical attributes of the fruit, such as its size, length, diameter, and firmness, as well as the quantity and weight of fruits produced, improved when NPK humate was applied to Le-Conte pear trees, especially at a high rate. Moreover, fruit juice’s total sugar content, soluble solids content, and soluble solids content/acid ratio all increased as acidity dropped^31^. When commercial humic substances were sprayed foliar on table grapes, Ferrara and Brunetti^32^ and Sánchez-Sánchez et al.^33^ observed a comparable response. Both the chlorophyll content and vegetative growth had significantly improved, according to their reports. Moreover, the yield increased significantly due to a significant increase in the berry and bunch weight and length. Titratable acidity fell as total soluble solids and the total soluble solids/acid ratio rose. Following the application of humate, they observed that the trees’ absorption of mineral nutrients—which are critical for fruit production and result in larger fruit—was most likely the source of the increase in fruit size. Moreover, humic acids enhance plant growth by improving photosynthesis, root development, and cell membrane permeability, facilitating the uptake of essential nutrients^34,35^. The possible hormone-like activity of the humic substances—which could include auxins, gibberellins, and cytokinins—should also be taken into account^36^. Gibberellin also improves fruit firmness by increasing the number of cells in the fruit, which increases the ratio of cell wall to cell volume. Potassium is necessary for many fundamental physiological functions, such as the synthesis of proteins, the generation of sugars and starches, cell division and development, and the flavor and color of fruits. Applying potassium humate helps enhance the absorption of potassium^37^.

Regarding the advantages of H_2_O_2_ spraying for Le-Conte pear fruits and the incidence of fire blight, Hafez et al.^5^ discovered that spraying H_2_O_2_ on Le-Conte pear trees greatly enhanced the fruit’s diameter, firmness, weight, length, and yield per tree. H_2_O_2_ foliar application not only markedly decreased the incidence percentage of fire blight in flowers or leaves and fruits of pear trees compared to control treatment but also improved the fruit quality in terms of higher fruit total soluble solids and reduced fruit acidity. Khandaker et al.^38^ connected the increased growth of wax apple trees sprayed with H_2_O_2_ to a significant increase in photosynthesis rates, stomatal conductance, and leaf transpiration. This growth was observed in terms of leaf dry matter and chlorophyll content, as well as a marked increase in fruit size, total soluble solids, and total sugar content, and yield. The greater yield is also ascribed to the function of H_2_O_2_ as a signaling molecule, which regulates photosynthesis, respiration, translocation, and transpiration^39^. The reduced incidence of fire blight may be related to H_2_O_2_'s capacity to slow down the rate of bacterial growth^40^. Also, plants produce reactive oxygen species (ROS) as a defense mechanism against bacteria such as *Erwinia amylovora*^41^. Hydrogen peroxide, one of the most significant and harmful of these free radicals, is distinguished by its high stability, capacity to pass through cell membranes and walls, and high reactivity with proteins, lipids, and nucleic acids^42^. The bacteria also promote the synthesis of specific enzymes that counteract these free radicals^43^. *Erwinia amylovora* and plants interact in one of two ways: either the plant cells detect the bacterial cells and react with an oxidative burst and a hypersensitive response, which lowers the pathogen populations and stops tissue colonization^44^, or the bacterial cells are able to avoid or reduce both the release of ROS and the hypersensitive response, which kills plant cells and advances within host tissues, resulting in the characteristic necrosis of fire blight disease^45^. As a result, applying hydrogen peroxide externally may strengthen the plant’s resistance to bacteria. The presence of H_2_O_2_ in the fruits, which accelerates the ripening process, and its ability to support cellular development and regulate cell expansion may account for the improved chemical properties of the pear fruits^46^.

The stimulating effect of spraying irradiated licorice extract on Le-Conte pear fruit characteristics was also found by Abd El–Hamied and El-Amary^4^ who discovered that applying licorice extract to Le-Conte “pear” substantially boosted the fruit’s total soluble solids, total sugar, vitamin C content, and fruit length, diameter, volume, weight, and number per tree. On the other hand, such treatments significantly decreased the number and percentage of fire blight infected fruits as well as fruit juice total acidity. Similar findings were reported in the studies conducted by Hasan and Kader^47^ on Sawa, Anbhu et al.^48^ on Bhagwa, and Nooruldeen et al.^49^ on Salemy pomegranate cultivars. Pomegranate trees were sprayed with licorice extract at varying rates, which significantly improved the fruit’s yield characteristics as well as its physical and chemical characteristics. Spraying irradiated licorice extract enhanced the benefits of reducing the percentage of sunburned and cracked fruits and boosting vegetative development, nutritional status, fruit physical and chemical properties, leaf yield, and nutritional content. This improvement was comparable to that of Ahmed et al.^50^, who found that irradiated licorice extract sprayed on red globe grapevine significantly improved vegetative growth as well as the percentages of NPK and total chlorophyll in the leaves. Moreover, it reduced the number of sunburned bunches and the percentage of berry acidity while increasing berry volume, weight, TSS%, anthocyanin content, bunch weight, and yield.

The improved physical and yield characteristics are due to the magic ingredient, mevalonic acid. Mevalonic acid stimulates the synthesis of gibberellin, which promotes increased cell division and expansion in the fruit, increasing its weight, size, and yield. Additionally, it is rich in growth phytohormones, minerals, vitamins, amino acids, carbohydrates, and salts. These nutrients support root and vegetative growth in addition to cell division and elongation. These elements, in addition to the fruits’ enhanced ability to take in water and nutrients from the soil, raise the yield parameters^51,52^. A possible explanation for the observed decrease in fire blight following the application of irradiated licorice extract is an increase in the synthesis of phenolic compounds. These compounds are known to impede the proliferation of pathogens and reinforcing the plant cell wall, enabling plants to respond promptly to pathogen assaults^53,54^. Furthermore, licorice extract enhances the plant’s resistance to infections by altering the plant’s responses to the threat through the use of salicylic acid and ethylene^55^. The reduced total acidity, however, can result from acid exhaustion in numerous physiological functions, such as respiration. The increased fruit total soluble solids after foliar licorice spraying may be due to increased leaf area and chlorophyll content, which in turn improved the uptake of soil nutrients and the synthesis of carbohydrates^56^.

When it comes to medicinal plants, radiation is a delicate process because different radiation doses have different effects on plants and asymmetric effects on the same plant in terms of content, especially volatile components, making some components disappear and others appear. In the case of licorice, gamma-irradiation with 10 kGy does not cause any volatile compounds to disappear; instead, it causes the appearance of new compounds like benzaldehyde and a noticeable increase in the amount of many other compounds like indole. Once more, licorice exposed to a 10 kGy dose of radiation showed a 12% increase in the maximum yield of its volatile compounds and the highest content of all the compounds present when compared to non-irradiated licorice^27^.

## Conclusions

This study demonstrated that application of a foliar spray of potassium humate mixed with hydrogen peroxide or irradiated licorice extract effectively enhanced the productivity and quality of Le-Conte pear fruits in terms of both physical and chemical characteristics while reducing fire blight-infected fruits. Spraying high levels of potassium humate and irradiated licorice extract produced the best results in terms of fruit quality; however, high levels of hydrogen peroxide and potassium humate resulted in the greatest reduction of fire blight infection and an improvement in the majority of fruit quality attributes. Thus, these cost-effective and environmentally friendly foliar treatments represent a promising approach to enhance orchard productivity and fruit quality. Further research is recommended to refine application protocols and assess their long-term benefits in various environmental conditions.

## Materials and methods

The study was conducted throughout two consecutive seasons in 2022 and 2023 on fifteen-year-old Le-Conte pear trees that were uniformly vigorous, budded on Pyrus communis rootstock, and planted in a private orchard at a distance of 70 km from Cairo on the Cairo Alexandria desert road, Egypt (30° 13′ 31.4″ N 30° 39′ 09″ E). The owner of the farm granted us permission to conduct the study, and we did so in compliance with local laws. The trees were grown using a drip irrigation system, and the planting space was 4 × 4 m. The trees were uniform in vigor and received common horticultural practices with the addition of half the amount of potassium sulfate fertilization. The tested trees were sprayed to drip point with potassium humate at (0, 1, and 2%) in 21 February, 21 March, 21 April, 21 May, and 31 June, while hydrogen peroxide at (0, 1, 2 ml/L) and gamma-irradiated licorice at (0, 4, 8 g/L) were sprayed at 15 February, 1^st^ March, 15 March, 15 April, and 15 May for both seasons. The method used for extracting dried licorice root was as described by Ahmed et al.^50^. Licorice extract was irradiated with a 10 KGy dosage at the Egyptian Atomic Energy Authority’s National Centre for Radiation Research and Technology in Cairo, Egypt, using a Gamma Cell (60Co) at dose rate 0.773 and 0.678 for February 2022 and 2023, respectively. The irradiated licorice extract constituents were as follow total phenols (23.2%), total flavonoids (15.7%), amino acids (9.3%), N (2.7%), P (2.5%), K (1.89%), Fe (50 ppm), Zn (63 ppm), and Mn (7 ppm). The physical and chemical properties of the soil during two seasons (2022 and 2023) were shown in Table 6. It was sandy loam soil, which is good for pear tree growth. The pH of the soil was 8 in both seasons, which is near the pH range of 6–7 that is ideal for raising productivity. Notably, soil properties improved slightly in 2023 compared to 2022, with a decrease in soil salinity (EC, Cl⁻, and Na) and an increase in certain nutrients like calcium and potassium, indicating improved soil quality for agriculture.

**Table 6:** Soil physical and chemical properties.

Season	Soil texture	Sand	Clay	Silt	pH (1:2.5)	EC (dS/m)	Cl^-^	SO_4_^2-^	Ca^2+^	Mg^2+^	Na^+^	K^+^	HCO_3_^-^
		(%)					(mmol/L)						
2022	Loamy sand	83.5	4.0	12.5	8.01	9.99	89.1	10.8	24.0	19.2	51.2	1.43	1.49
2023	Loamy sand	83.0	4.5	12.5	8.00	8.81	73.3	11.9	26.5	16.1	49.1	1.60	1.66

This experiment used a fully randomized block design with 15 treatments and three replicates of three trees each. To eliminate surface tension, each tree got 3 L of the applied solution plus 5 cm per liter of 20, except for the control treatment, which received water and 5 cm per liter of 20. The trial contained the following fifteen treatments: Control (no foliar spray), K1 (potassium humate at 1%), K2 (potassium humate at 2%), H1 (hydrogen peroxide at 1 ml/L), H2 (hydrogen peroxide at 2 ml/L), L1 (gamma-irradiated licorice at 4 g/L), L2 (gamma-irradiated licorice at 8 g/L), K1H1, K1H2, K1L1, K1L2, K2H1, K2H2, K2L1, and K2L2.

### Fruit quality parameters

1. Fruit physical characteristics: size (cm^3^), height (cm), diameter (cm), shape index, and firmness (N; Newton) were measured on 15 fruits harvested from each duplicate tree.
2. Yield characteristics: harvesting achieved on (15th August for each season), fruit weight (g), fruit number, and yield (Kg/tree) were recorded. In March and April of each season, a visual inspection of pear fruits was conducted, during which the number of fruits affected by fire blight was recorded, and the corresponding percentages were calculated.

Fruit chemical properties: according to AOAC^57^, the Abbe digital refractometer was used to measure the percentage of total soluble solids (TSS). According to AOAC^57^, total acidity was calculated as citric acid by titration with a 0.1 N. NaOH solution and phenolphthalein as an indicator. The values were computed as grams per 100 milliliters of juice. The total soluble solids/acid ratio was obtained using the following equation: Total soluble solids/acid ratio = TSS/ total acidity. The method described in AOAC^57^ was used to determine the total juice sugar: total sugars (g/100 g FW). This involved naturalizing 25 ml of juice sample to a pH of 7.5 to 8.0 with 1 N NaOH, adding 2 ml of lead acetate and a few drops of potassium oxalate, and diluting the mixture. Then, 5 g of citric acid was added to the filtrate, neutralized using phenolphthalein as an indicator, and neutralized with 20% NaOH until a pink hue was achieved. The titration’s end point was colorless. Sugars were calculated using the previously described methodology by Ali et al.^58^. To turn non-reducing sugars into reducing sugars, a sample of fruit pulp treated with 25% lead acetate and 20% potassium oxalate was hydrolyzed with HCl and left overnight. The aliquot that had been hydrolyzed by HCl was then titrated against Fehling solutions after being neutralized with 0.1 N NaOH. The fruits’ potassium content was determined by flame photometry, and their zinc and iron contents were determined by atomic absorption spectrometry^59^.

### Statistical analysis

This study employed a randomized full-block design with three replicates. The current data was statistically analyzed by Snedecor and Cochran^60^. The new L.S.D. values were used to compare averages at the 5% level. According to McLain^61^, the MSTAT program was used to compute the collected data.

## Data Availability

The data that support the findings of this study are not openly available due to reasons of sensitivity and are available from the corresponding author upon reasonable request.
